# Epidemiology of Eventration: Predicting Prevalence

**DOI:** 10.7759/cureus.90231

**Published:** 2025-08-16

**Authors:** Elena LeCompte, Schafer Paladichuk, Sadie Walter, Jocelyn Larsen, Ronald Walser, Wade W Justice

**Affiliations:** 1 Clinical Medicine, Pacific Northwest University of Health Sciences College of Osteopathic Medicine, Yakima, USA; 2 Anatomy, Pacific Northwest University of Health Sciences College of Osteopathic Medicine, Yakima, USA

**Keywords:** chest x-ray, community medicine, community medicine & public health epidemiology, eventration of diaphragm, incidence and prevalence, incidental pulm, public health epidemiology

## Abstract

Introduction

Diaphragmatic eventration (DE) is characterized by an abnormal elevation of the diaphragm. While typically asymptomatic and incidentally discovered on imaging, DE can pose diagnostic challenges due to its ability to mimic other pathologies. We hypothesize that the prevalence of DE is underreported, owing to the lack of existing reliable literature exploring the true epidemiology of DE.

Methods

A retrospective review of chest X-rays (CXRs) was conducted using the MIT Medical Information Mart for Intensive Care, Fourth Edition (MIMIC-IV) database. Preoperative CXRs were screened for evidence of DE, defined as a >2.5-cm elevation of one hemidiaphragm on the frontal view and confirmed on lateral imaging. Diaphragmatic heights were measured using DICOM imaging software, and inter-rater reliability was assessed among four reviewers, including a board-certified radiologist, to confirm visualization. A larger review of CXR data was performed by the board-certified radiologist to assess for a possible true prevalence of DE.

Results

Of the 279 formally reviewed cases by the diagnostic radiologist, 196 (70.3%) met the inclusion criteria. Among those, 46 (23.5%) patients exhibited radiographic evidence of DE, significantly exceeding previously reported prevalence rates.

Conclusion

Our findings suggest that DE may be more common than previously reported. Unrecognized DE can lead to unnecessary workups for other pathologies. Recognizing DE as a frequently seen normal anatomical variation can reduce diagnostic uncertainty. Further research is needed to refine the epidemiology and clinical relevance of DE.

## Introduction

Diaphragmatic asymmetry on CXR is often encountered in clinical medicine. It has a plethora of etiologies, including multiple pathologic conditions involving the lung, the phrenic nerve, the abdominal contents, and even the diaphragm itself [1]. One of the known diaphragmatic conditions that often causes asymmetry is diaphragmatic eventration (DE). This condition has been known and discussed in the literature for nearly 100 years [2]. DE refers to the abnormal contour of the diaphragmatic dome with no disruption to the diaphragmatic continuity [1,3]. While a formal height definition for DE is not universally recognized, the normal right hemidiaphragm is, on average, less than 2 cm higher than the left [4]. The height difference has also been described by the “thoracic vertebral level” determination, and the right diaphragm was found to be 0.5 levels higher than the left one in patients with normal pulmonary function [5]. The right hemidiaphragm tends to be elevated in comparison to the left due to the liver occupying the right upper abdominal quadrant [4].

DE has been categorized into two variants: congenital and acquired. Typically, congenital DE only affects a segment of the hemidiaphragm, whereas acquired DE results in paralysis/weakness of the diaphragm or diaphragmatic retraction, leading to the entire hemidiaphragm being affected [1]. DE is often identified incidentally on chest X-ray (CXR) or computed tomography (CT) scans [1,3,6,7]. Congenital DE is often the result of incomplete muscularization of the diaphragm, with a thin membranous sheet replacing the normal muscle [1,6,7]. Acquired cases are more common [3]. Congenital DE is frequently seen in the anteromedial portion of the right hemidiaphragm, while acquired eventration is frequently seen in the left hemidiaphragm [1,3].

The less rigorously defined nature of DE greatly limits accurate reporting of its occurrence; the prevalence of DE is suggested to be as low as less than 0.05%, with a predominance in males, and has been reported to most commonly affect the left hemidiaphragm [3,8-11]. We wondered if the true prevalence of DE is potentially significantly higher than reported. Diaphragmatic asymmetry can be an important CXR finding and may be associated with a number of differential diagnoses, ranging from benign to life-threatening conditions [1,3,8,11]. Thus, defining the true epidemiology of diaphragmatic asymmetry is crucial in mitigating potential diagnostic dilemmas during radiological interpretation [11].

We sought to investigate a commonly encountered congenital DE variant, which is unilateral and partial, affecting the front half of the right diaphragm [11]. This variant, which does not need further investigation, can often be confused with the other causes of diaphragmatic asymmetry [11]. Often, diaphragmatic asymmetry can lead to further radiological and/or clinical work-up to exclude subpulmonic fluid, subdiaphragmatic masses, phrenic nerve paralysis, and numerous causes of right pulmonary atelectasis [11]. Congenital DE is often underreported and not recognized as a normal variant [1,3,8,11]. Our clinical experience was suggestive of this type of DE being much more common than 1%. We hypothesize that the prevalence of DE is underreported, owing to the lack of existing reliable literature exploring the true epidemiology of DE [11].

## Materials and methods

The Institutional Review Board (IRB) of the Pacific Northwest University of Health Sciences College of Osteopathic Medicine (PNWU-COM) deemed this project as exempt (IRB #25-011). It was initially approved on April 30, 2025. All data were deidentified before publication, and, thus, the need for consent was waived.

A retrospective chart review was conducted to obtain de-identified patient CXRs along with official radiology reads. Lead researchers became credentialed with a signed waiver to gain access to the MIT Medical Information Mart for Intensive Care (MIMIC) Fourth Edition databases. The MIMIC database is a large publicly available dataset of 364,627 deidentified patient charts. The following subsections were utilized: MIMIC-IV, MIMIC-IV CXR database, and MIMIC-IV free-text clinical notes [12-15]. The reports and images were collected from the Electronic Health Records of the Beth Israel Deaconess Medical Center from 2011 to 2016. Charts were scanned looking for CXRs requested as part of a pre-operative test in an attempt to limit inclusion of cases that had pre-existing conditions that might cause diaphragm asymmetry. CXRs were then downloaded and viewed within the Digital Imaging and Communications in Medicine (DICOM) and Bee DICOM imaging software from the MIMIC-IV CXR database [15]. Heights of the diaphragms were measured with the DICOM software using the measuring function, and heights (in cm) were recorded.

**Figure 1:**
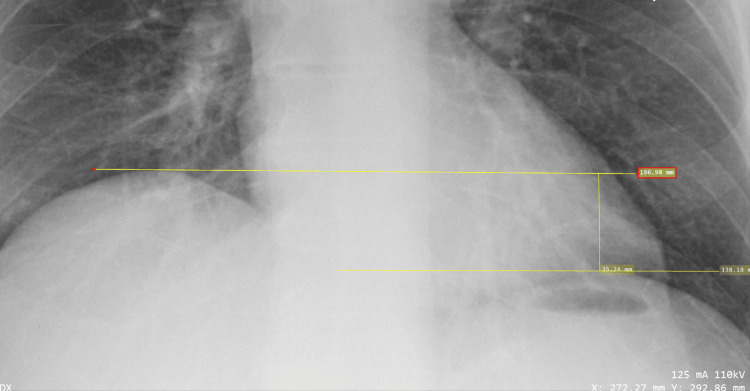
Frontal CXR projection obtained from the MIMIC-IV CXR database utilizing DICOM software and the line technique with evidence of DE on the right side. Yellow lines indicate parallel lines placed at the apex of both hemidiaphragms. Hemidiaphragm height differences (vertical yellow line) measured at 3.52 cm. CXR from the MIMIC-CXR Database v2.0.0, a publicly available dataset developed by Johnson et al. at the Massachusetts Institute of Technology. Image used under a Creative Commons Attribution 4.0 International License (CC BY 4.0). Source: Johnson et al. [14] CXR, chest X-ray; DE, diaphragmatic eventration; MIMIC-IV, MIT Medical Information Mart for Intensive Care, Fourth Edition

**Figure 2:**
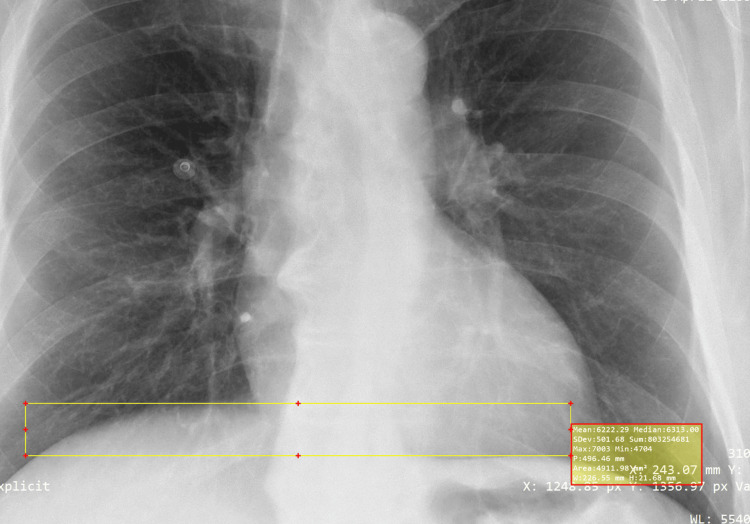
Frontal CXR projection obtained from the MIMIC-IV CXR database utilizing the DICOM Bee software and the box technique while assessing for evidence of DE. Yellow parallel lines are placed at apex of both hemidiaphragms. Hemidiaphragm height differences measured at 2.4 cm. CXR from the MIMIC-CXR Database v2.0.0, a publicly available dataset developed by Johnson et al. at the Massachusetts Institute of Technology. Image used under a Creative Commons Attribution 4.0 International License (CC BY 4.0). Source: Johnson et al. [14] CXR, chest X-ray; DE, diaphragmatic eventration; MIMIC-IV, MIT Medical Information Mart for Intensive Care, Fourth Edition

**Figure 3:**
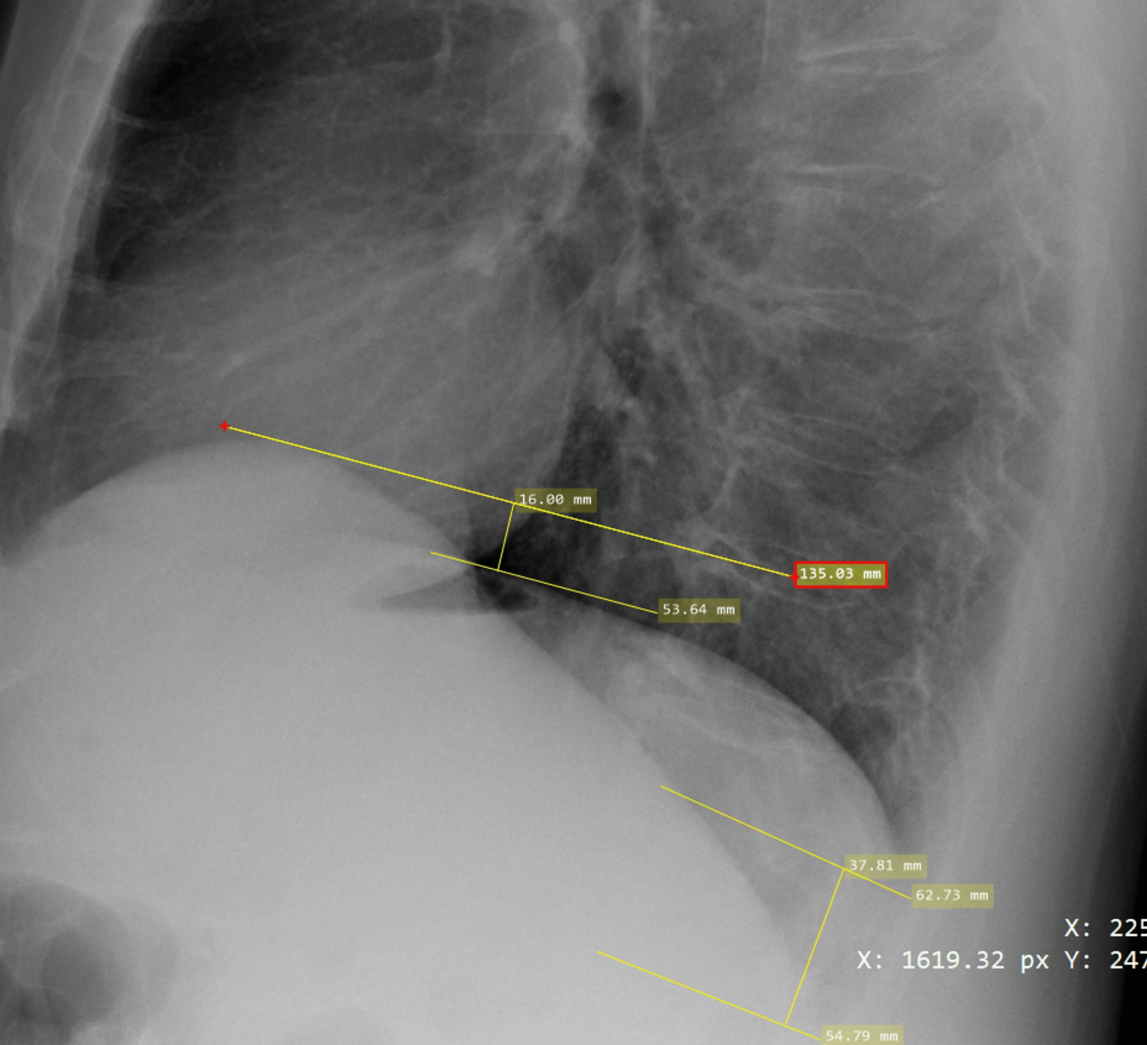
Lateral CXR projection obtained from the MIMIC-IV CXR database with evidence of DE on the right hemidiaphragm. Anterior parallel lines demonstrate the measurements of the anterior height differences, while posterior lines demonstrate the measurements of posterior height differences. Perpendicular lines represent the height distance (in cm), where the anterior measurement was 1.6 cm and posterior measurement was 3.78 cm. CXR from the MIMIC-CXR Database v2.0.0, a publicly available dataset developed by Johnson et al. at the Massachusetts Institute of Technology. Image used under a Creative Commons Attribution 4.0 International License (CC BY 4.0). Source: Johnson et al. [14] CXR, chest X-ray; DE, diaphragmatic eventration; MIMIC-IV, MIT Medical Information Mart for Intensive Care, Fourth Edition

Heights of the diaphragm were measured on the lateral and frontal images to evaluate for DE. Frontal projection heights were determined by utilizing either the line function within the DICOM software (Figure 1) or square function within the Bee DICOM software (Figure 2). Parallel lines were placed at the apex and along the contour of both the left and right hemidiaphragms. A perpendicular line was then placed between the two parallel lines, which provided hemidiaphragm height differences (Figures 1, 2). Once it was deemed a positive case on the frontal projection, measurements were taken on the lateral projection to confirm or refute. Lateral projection heights were measured at two places. The first place was the apex of the two hemidiaphragms. The right hemidiaphragm, which is typically not obscured by the heart silhouette, is measured at the apex with a line that is roughly perpendicular to the vertebral bodies. The left hemidiaphragm, which is typically obscured by the heart silhouette, is measured with a parallel line to the other where the diaphragm border meets the cardiac silhouette (Figure 3). Again, these two lines are parallel to each other, with a perpendicular line place between them to determine the height difference between the diaphragms. A second measurement was taken where the two hemidiaphragms meet the ribs posteriorly at the posterior sulcus. Two parallel lines were placed at the depth of the posterior sulcus perpendicular to the vertebral bodies. These two lines were not always parallel to the anterior apex lines. A perpendicular line was placed between the two parallel lines, or a box shape was placed along the diaphragms to create a perpendicular line, which represented the difference in diaphragm heights (Figure 3). If the right hemidiaphragm was elevated at both the frontal apex measurement and posterior sulci measurement, this could not be recorded with any certainty as DE since this could also represent pathologic complete diaphragm elevation. This would also be the same for elevation of the left hemidiaphragm.

An initial proof-of-principle study was conducted. Four researchers (three medical students and one board-certified radiologist) examined a small cohort of patients [11]. All four researchers provided measurements (in cm) of the patients’ hemidiaphragms including anterior and lateral views. Patients were identified with possible DE if there was a >2-cm difference between the height of the hemidiaphragms on the frontal projection [11]. This was confirmed or refuted by the appearance of the diaphragms on the lateral projection [11]. Cohen’s K-coefficient was then conducted for statistical analysis within Microsoft Excel (Microsoft Corp., Redmond, WA).

The proof-of-principle cohort reassured the study team that observations of the hemidiaphragms utilizing CXR can be done effectively at the medical student level and board-certified level. This, in turn, lead to a full retrospective study. An initial statistical power calculation was conducted to determine the cohort size needed to obtain a power of 0.8. The statistical test (Sample Size Calculator, StatsKingdom.com) was set as regression, alpha set to 0.05, and sample size set to 76, and it was calculated as a power of 0.8018. For this part of the study, we again chose to analyze CXRs of pre-operative patients, and the hemidiaphragm height was adjusted to >2.5 cm for positive DE. A board-certified radiologist then read the CXRs from this cohort. All presumed positive DE cases on the frontal projection (Figure 1) were then confirmed or refuted by the appearance on the lateral projection (Figure 2). Additional data included demographic information (age, sex) with standard deviations (SDs).

## Results

For the initial proof-of-principle study, a cohort of 105 patients was examined by the research team. Inter-rater reliability was significant with a Cohen’s K coefficient of 0.71 when identifying DE as >2 cm on the anterior and lateral CXRs, indicating substantial agreement. This initial study demonstrated findings suspicious for DE in 30 (28.6%) patients in the proof-of-principle study cohort.

When assessing the larger cohort, a total of 279 patients were identified through the retrospective chart review. Of the 279 patients, 83 (29.7%) were excluded due to one or more of the following reasons: the CXR was not taken preoperatively, CXR was not a two-view study, the image file was inaccessible, or the diaphragmatic contours could not be clearly identified on CXR. This left the patient cohort with 196 patients (Table 1). Of the studied cohort, 46 (23.5%) had evidence of eventration on their CXRs, which is further divided by sociodemographic factors in Table 2.

**Table 1:** Sociodemographic characteristics of the study population Gender is represented by N (%) of the total population. Age is represented as mean (SD). N, total study population; SD, standard deviation

Sociodemographic characteristics		N (%)/mean (SD)
Gender	Female	80 (40.8)
	Male	116 (59.2)
Age (years)	Entire cohort	54.9 (14.7)
	Female	56.9 (14.1)
	Male	53.5 (15.0)

**Table 2:** Prevalence of DE based on the sociodemographic characteristics of the participants DE, diaphragmatic eventration

Prevalence of DE based on sociodemographic characteristics			DE		Total
			Yes	No	
Total		N	46	150	196
		%	23.5%	76.5%	100%
Gender	Female	N	17	63	80
		%	21.3%	78.7%	100%
	Male	N	29	87	116
		%	25.0%	75.0%	100%

## Discussion

The true prevalence of DE is unknown [11]. Many authors have estimated it to be rare, while many have stated its prevalence as less than 1% [1,3,8-11]. We have shown that the likely prevalence of congenital DE is common and may affect a quarter of all adult patients [11]. Managing DE mostly depends on severity and etiology [1,3]. Often, the diaphragmatic asymmetry is due to other causes such as lung atelectasis, pleural fluid, or subdiaphragmatic masses, and it is important to identify DE from these pathologic conditions. While many patients with DE present asymptomatically, those who present with symptoms, mild or severe, often require surgical intervention such as surgical plication of the diaphragm [3]. If left untreated in a patient with symptoms, it may develop into respiratory failure, recurrent pneumonia, and dyspnea that is not due to other disease processes such as heart failure [3]. Congenital DE accounts for most of the reported symptomatic cases and almost always requires surgical intervention [1,9,10,16].

There are several limitations to this study. When utilizing CXRs, patient-specific factors such as body habitus, posture, and positioning and differences in respiratory effort during imaging might have resulted in variability when measuring the heights of the hemidiaphragms. At times, it was difficult to confidently identify the diaphragm positions on the lateral as the posterior sulci were sometimes not optimally visualized. Also, we could not say with confidence these patients had any pathologic condition explaining their diaphragmatic asymmetry, but assumed it was less likely due to it being a pre-operative CXR. Additionally, we used different versions of the DICOM software to measure the diaphragm height differences. The line function required us to draw parallel lines along the dome of each diaphragm and then draw a perpendicular line between them to measure the height difference. The box version allowed us to place a box shape over both diaphragms to determine the height difference with one keyboard maneuver. An example of each technique in illustrated in the figures. Lastly, another limitation was the reliance of our data derived from a single cohort, which may not fully represent diverse populations or account for other clinical variables influencing DE. While the study provided a foundation on the prevalence of DE, the findings were based on 196 clinical cases from a single hospital, which may limit the broad significance of the conclusions.

## Conclusions

In conclusion, this study aimed to provide a more accurate estimate of the prevalence of DE. Our findings suggest that DE may be far more prevalent than previously reported, warranting greater clinical recognition. Perhaps, the reported abnormal diaphragmatic asymmetry should be increased from 2.0 cm. Although often asymptomatic and dismissed as incidental, DE can mimic more serious pathology and lead to unnecessary investigations. Accurate identification and reporting of this anatomical variant are essential to reduce diagnostic confusion and avoid inappropriate workup. As a frequently encountered finding on CXRs, DE should be acknowledged not as a rare anomaly but as a relatively common and benign variation of normal anatomy. Further multicenter studies are necessary to validate these findings across diverse populations and reinforce the importance of recognizing DE when performing routine radiological evaluation of the chest.
